# Mechanisms of drug resistance of pancreatic ductal adenocarcinoma at different levels

**DOI:** 10.1042/BSR20200401

**Published:** 2020-07-30

**Authors:** Jiali Du, Jichun Gu, Ji Li

**Affiliations:** Department of Pancreatic Surgery, Huashan Hospital, Fudan University, Shanghai, P.R.China

**Keywords:** Cellular immunotherapies, Drug resistance, Gemcitabine, Molecular targeted therapy, Pancreatic ductal adenocarcinoma (PDAC)

## Abstract

Pancreatic ductal adenocarcinoma (PDAC) is the fourth leading cause of cancer-related death worldwide, and the mortality of patients with PDAC has not significantly decreased over the last few decades. Novel strategies exhibiting promising effects in preclinical or phase I/II clinical trials are often situated in an embarrassing condition owing to the disappointing results in phase III trials. The efficacy of the current therapeutic regimens is consistently compromised by the mechanisms of drug resistance at different levels, distinctly more intractable than several other solid tumours. In this review, the main mechanisms of drug resistance clinicians and investigators are dealing with during the exploitation and exploration of the anti-tumour effects of drugs in PDAC treatment are summarized. Corresponding measures to overcome these limitations are also discussed.

## Introduction

Pancreatic ductal adenocarcinoma (PDAC), accounting for approximately 90% of pancreatic cancers, is one of the most invasive malignant tumours. In the United States, the numbers of estimated new cases of pancreatic cancer and deaths in 2018 are 55,440 and 44,330, respectively, and the 5-year survival rate from 2008 to 2014 is 8.5% [1]. The poor prognosis is closely related to the limited symptoms that are of diagnostic value and significant biomarkers that can be used for early screening as well as early metastasis with aggressiveness [2]. Surgery remains the only chance for a possible cure of pancreatic cancer [3]. As a result of the aforementioned limitations, only 15–20% of patients with PDAC are eligible for potentially curative surgery [4], while a large proportion of patients are diagnosed with either metastatic or locally advanced tumours [5], emphasizing the importance of therapeutic strategies except surgery. Moreover, surgery alone is also ineffective for qualified patients because more than 90% of them experience relapse and die of the disease after potentially curative surgery followed by no additional therapy [6]. Hence, adjuvant treatment approaches have been comprehensively explored during the past several decades. Various therapeutic regimens, including classical cytotoxic drug as well as chemotherapy based on it, targeted therapeutic drugs, and emerging cellular immunotherapy, have been used for treating PDAC, and several are still under study. However, only few of them distinctly and enduringly improve the prognosis of patients with PDAC due to serious drug resistance. In this review, we summarize the mechanisms of drug resistance at different levels, including the body, tumour microenvironment (TME) and cells in it, and molecules, which currently impede drugs from playing its due role in the treatment of PDAC and are puzzling clinicians and investigators at the same time. Some response measures under study are also introduced.

## Chemotherapy based on gemcitabine

In 1997, Burris et al. demonstrated that gemcitabine-treated patients showed a better clinical benefit compared with those who were treated with 5-fluorouracil (5-FU) [7]. Gemcitabine has been a cornerstone of PDAC treatment for patients with a quite good performance status [8]. Because of the modest clinical benefits of gemcitabine monotherapy on patient survival (response rate of approximately 10%) [9], new combination strategies have been explored, among which the most commonly used first-line regimens nowadays are FOLFIRINOX (folinic acid, 5-FU, irinotecan, and oxaliplatin) and AG (the doublet of gemcitabine and nab-paclitaxel).

### Chemoresistance at the TME levels

#### Desmoplasia and hypovascularity

**Figure 1:**
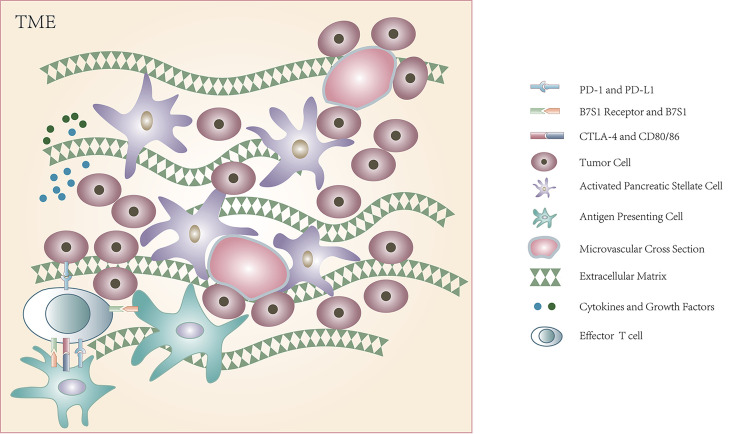
Hypovascular tumour microenvironment with dense stroma of PDAC

The stroma in PDAC is extremely heterogeneous and comprises cellular and acellular components including fibroblasts, myofibroblasts, immune cells, blood vessels, extracellular matrix (ECM), and some soluble proteins (Figure 1) [10]. The dense desmoplastic stroma in pancreatic cancer, in addition to its hypovascular nature, mostly due to the compression exerted by ECM rigidity [10], has been regarded as a physical barrier for therapeutic drug delivery. Several factors such as pancreatic stellate cell (PaSC), secreted protein acidic and rich in cysteine (SPARC), hyaluronan, and Hedgehog (Hh) signalling pathway as well as some cytokines and growth factors have been shown to contribute to the dense stroma and poor drug delivery, such as platelet-derived growth factor (PDGF), insulin-like growth factor 1 (IGF-1), and endothelin-1 (ET-1), which could contribute to the proliferation of PaSC, transforming growth factors (TGFs), and fibroblast-growth-factor-2 (FGF2), which could stimulate matrix synthesis, and TGFβ1, tumour necrosis factor-α (TNFα), promoting the activation of PaSC [11–15]. Apart from that, different from several other solid tumours, PDAC cells produce not only angiogenic but also anti-angiogenic factors such as angiostatin, endostatin, and pigment epithelium-derived factor strongly inhibiting angiogenesis [16]. Thus, diversified strategies aiming to eliminate the fibrotic stroma and reversing the hypovascular state for the enhanced drug delivery to tumour cells have been studied. Some of the therapies targeting stroma led to a reduction in the abundant stroma, inducing enhanced drug delivery and longer progression-free survival (PFS) [12,17], while some trials targeting the Hh signalling pathway showed a noticeable increased survival rate of progressive disease [3]. As a result, it seems that some components of stroma in PDAC contribute to the formation of a physical barrier for drug delivery, making tumour difficult to be intervened, while others could prevent tumour cells from progression and metastasis to some extent [3,18,19].

#### Hypoxia

Hypoxia is a common feature of several solid tumours in which pancreatic cancer has been discovered to be the most hypoxic [20]. The main reason for the reduced PDAC oxygenation is the hypo-vasculature that could not deliver sufficient oxygen to tumour tissue via the bloodstream [16]. Through slowing down the cell cycle, hypoxia could enhance resistance to cytotoxic drugs, which inhibit tumour cells by interrupting their DNA synthesis and proliferation. Moreover, in a hypoxic microenvironment, hypoxia-inducible factor-1 (HIF-1) could act as a regulator initiating adaptive responses of pancreatic cancer cells to ensure their survival. In this process, some proapoptotic proteins are down-regulated, like Bid and Bax, while some are up-regulated, such as BNIP3, Noxa or NIX, and some antiapoptotic proteins could also be up-regulated like Bcl-X_L_, indicating the dichotomy in the ability of hypoxia to modulate apoptosis [21–28]. However, in PDAC, the proapoptotic protein BNIP3 induced by HIF-1 is silenced both via epigenetic changes and clonal elimination of BNIP3-positive cells, indicating the antiapoptotic role of HIF-1 because proapoptotic pathways like BNIP3 are most likely down-regulated [21–23]. It has been found that the activation of hypoxia-inducible pathways, including phosphoinositide-3 kinase/protein kinase B (PI3K/Akt) and nuclear factor kappa-light-chain-enhancer of activated B cells (NF-κB) signalling pathways, could contribute to the resistance to gemcitabine [29].

### Chemoresistance at the molecular levels

#### Transmembrane transportation of gemcitabine dependent on human equilibrative nucleoside transporter 1

Gemcitabine, 2′,2′-difluorodeoxycytidine (dFdC), is a deoxycytidine nucleoside analogue, killing tumour cells by inhibiting DNA replication and thus blocking cell cycle progression [30]. Hence, it must be transported into the tumour cells before it works. Due to the property of hydrophilicity, the transportation of gemcitabine into tumour cells is highly dependent on human nucleoside transporters (NTs), whose biological activity is considered as a prerequisite for the biochemical efficacy of gemcitabine [31]. Human equilibrative nucleoside transporter 1 (hENT1) is the most primary NT mediating the uptake of gemcitabine, so hENT1-deficient tumour cells are strongly resistant to gemcitabine [32]. Moreover, the inhibition of hENT1 could produce the same effect.

#### Various enzymes influencing the metabolism of gemcitabine

The biological activities of gemcitabine are subject to the expression level and activities of several enzymes in cells. Once transported into tumour cells, dFdC is phosphorylated into dFdCMP and dFdCDP by deoxycytidine kinase (dCK), pyrimidine nucleoside monophosphate kinase (also known as UMP/CMP), and nucleoside diphosphate kinase successively [33]. In addition, dFdCTP could be incorporated into the new DNA chain in DNA replication, replacing dCTP and thus successfully killing tumour cells. *dCK* is the rate-limiting enzyme of the process of the metabolism of gemcitabine, of which the levels of expression in tumour cells are intimately correlated with the activation and inactivation of gemcitabine. *Ribonucleotide reductase (RR)*, supposed to catalyse the reduction reaction from NTPs to dNTPs, is a rate-limiting enzyme of the DNA synthesis pathway [34]. RR blocking reduces the dNTP pool ready for DNA replication in the nucleus of tumour cells, which is conducive to the incorporation of dFdCTP into the DNA chain [35]. The RR level in tumour cells is negatively correlated with the activity of inhibiting the DNA replication and blocking the cell cycle progression of gemcitabine. *Cytidine deaminase (CDA)*, which mostly contributes to the rapid clearance of gemcitabine in human body, could induce dFdC deamination via the removal of the -NH2 group from pyrimidine [34]. DFdU is not a suitable substrate for pyrimidine nucleoside phosphorylases and can only be excreted out of the cell.

### Limitations exerted by adverse drug effects

Because of the modest clinical benefits of gemcitabine monotherapy on patient survival (response rate of approximately 10%) [9], new combination strategies have been explored, among which the most commonly used first-line regimens nowadays are FOLFIRINOX (a therapy combination including folinic acid, 5-FU, irinotecan, and oxaliplatin) and the combination of gemcitabine and nab-paclitaxel. Although more significant outcomes than that of single agent gemcitabine have been achieved, early termination forced by accompanied worse toxicity and higher incidence of adverse effects [8], leading to the ineffectiveness of therapies, has become one of the bottlenecks in the achievement of expected clinical benefits of the combination chemotherapy agents. Thus, the two standard protocols are recommended for patients with an Eastern Cooperative Oncology Group (ECOG) performance status of 0–1 in the ASCO Clinical Practice Guidelines for metastatic pancreatic cancer [30].

FOLFIRINOX has been reported to induce a higher rate of grade 3/4 adverse events, including neutropenia (45.7% vs. 21%), fatigue (23.6% vs. 17.8%), vomiting (14.5% vs. 8.3%), diarrhoea (12.7% vs. 1.8%), and sensory neuropathy (9% vs. 0%), than gemcitabine [36]. Even in a group of patients with good performance status, these toxicity and adverse effects are still noticeable problems. Due to the high incidence of adverse events, there is not an optimization strategy for FOLFIRINOX therapy that could be widely accepted in several countries [9].

The comparison of the safety and efficacy of gemcitabine plus nab-paclitaxel and gemcitabine monotherapy was reported in a phase III trial. Compared with the gemcitabine group, the combination regimen substantially increased the OS, PFS, and response rate (RR), while the rates of peripheral neuropathy and myelosuppression increased, and treatment-related adverse events of grade 3 or higher including neutropenia (38% vs. 27%), leukopenia (31% vs. 16%), fatigue (17% vs. 7%), and peripheral neuropathy (17% vs. 1%) were reported more often in the nab-paclitaxel–gemcitabine group than in the gemcitabine group [37].

### Corresponding measures to override limitations for chemotherapy

To circumvent the resistance to gemcitabine derived from the physical barrier in PDAC, nano-carrier has emerged and the modifications of the gemcitabine such as prodrug has been designed to counter the resistance from process of gemcitabine metabolism [34]. The nano-carrier approach based on the kinds of nanoparticle has been reported as a promising measure, although its clinical application value is still under study, to reduce the dependence on hENT1 and enhance the permeability of gemcitabine, thereby increasing the concentration of gemcitabine in tumour cells, which has been demonstrated *in vitro* and *in vivo* [38,39]. A ‘prodrug’ is a biologically inactive form of a parent drug molecule exhibiting better delivery properties than the parent drug and requires an enzymatic or chemical transformation within the body to release the active drug entity. Modifications at 4-(N)- and 5′-positions of gemcitabine are the most studied [40]. It excels in providing (i) protection against deamination, (ii) better storage, (iii) prolonged release in the cell, and (iv) possible use in the case of deoxycytidine kinase deficiency [40].

To decrease the accompanied toxicity and adverse effect of two prominent regimens, FOLFIRINOX and the doublet of gemcitabine and nab-paclitaxel, the modifications of the FOLFIRINOX regimen to decrease the toxicity profile and keep a similar anti-tumour effect as the standard one at the same time have been tested [9,41]. Mahaseth et al. reported that modified FOLFIRINOX (discontinuation of the bolus 5-FU and administration of growth factors) has an improved safety profile, especially with respect to neutropenia, fatigue, and vomiting, with maintained efficacy in metastatic PDAC [42]. Stein et al. conducted the first prospective trial of modified FOLFIRINOX in metastasis PDAC, in which patients with untreated metastatic pancreatic cancer or locally advanced pancreatic cancer receiving modified FOLFIRINOX (irinotecan and bolus 5-FU reduced by 25%) showed a significantly decreased incidence of grade 3 or 4 neutropenia (12.2% vs. 45.7%), vomiting (2.7% vs. 14.5%), and fatigue (12.2% vs. 23.6%) compared with the historical standard FOLFIRINOX group [43]. At the same time, no significant difference in treatment efficacy was observed between modified FOLFIRINOX and full-dose FOLFIRINOX. Li et al. conducted the first prospective study to evaluate FOLFIRINOX in Chinese patients with metastatic pancreatic cancer and showed that modified FOLFIRINOX (oxaliplatin and irinotecan reduced by 15% and 25%, respectively, and omitting the fluorouracil intravenous bolus) was well tolerated in these patients, with an ORR of 32.5%, median OS of 10.3 months, and PFS of 7.0 months [9]. However, different from these promising results, de Jesus et al. have not found significant discrepancy in toxicity between patients with metastatic pancreatic adenocarcinoma treated with standard and modified FOLFIRINOX regimens, possibly due to patient selection and a higher dose reduction rate in the standard FOLFIRINOX arm. Moreover, the activities against metastatic pancreatic adenocarcinoma of the two regimens are similar in a retrospective study [41]. Therefore, the effective modification agent is still under exploration. Additionally, because only patients with an ECOG performance status of 0–1 were enrolled in these studies, it is unclear whether these modification modalities could be extended to patients with worse status, even if they have showed promising effects in these trials. Nonetheless, these studies have paved the way for the exploration of better modifications aiming to balance the toxicity and activity of chemotherapy drugs.

## Molecular targeted therapies

The main gene mutations in PDAC are KRAS genes, which are mutated in more than 90% of patients, and some tumour suppressor genes such as CDKN2, TP53, and SMAD4 in approximately 90%, 60%, and 55% of PDAC cases, respectively [8,44]. With other mutations accumulating in tumour development, all these mutational genes lead to the dysregulation of several kinds of signalling pathways in cancer and TME cells, among which some are up-regulated to a much higher level than in a normal body, while others are blocked, losing their biological activities. These aberrant signalling pathways play important roles in tumour proliferation, invasiveness, and metastasis and resistance to chemotherapy and radiotherapy [45]. Given that, targeted therapy has emerged as a possible approach to suppress tumour development, reverse the chemoresistance state, and thus produce survival benefits. Unfortunately, despite that several studies on targeted therapy for PDAC achieved promising outcomes in preclinical or clinical experiments, most of them failed in phase II/III trials, except erlotinib, an epidermal growth factor receptor (EGFR) inhibitor (Table 1) [8]. Additionally, the dense hypovascularized stroma remains to be a problem in limiting the drug accessibility. Moreover, significant biomarkers predicting the effectiveness of blocking of targets are yet to be demonstrated. Also, dose reduction induced by adverse effects and toxicities constantly stop these drugs as well as classical chemotherapies from embodying optimal anti-tumour effects [46].

**Table 1:** Selected clinical trials of molecule targeted therapy for pancreatic adenocarcinoma

Mechanism of action	Drug	Phase	Tumour of patients	*n*	Treatment group	Media survival (month)	One-year survival rate	PFS (month)	Statistically significantly improvement	Study
HER1/EGFR tyrosine kinase inhibitor	Erlotinib	III	Patients with unresectable, locally advanced, or metastatic pancreatic cancer	569	Erlotinib+gemcitabine; Placebo+gemcitabine	6.24/5.91 (*P*=0.038)	23%/17%(*P*=0.023)	3.75/3.55 (*P*=0.004)	YES	[47]
Monoclonal antibody targeting HER-2	Herceptin (brand name of Trastuzumab)	II	Patients with metastatic pancreatic cancer with 2+/3+ HER-2/neu expression	34	All patients received gemcitabine for 7 of 8 weeks followed by 3 of every 4 weeks, and Herceptin, 4 mg/kg loading dose, followed by 2 mg/kg/week.	7	19%	Not reported	Unclear	[48]
Monoclonal antibody targeting HER-2	Herceptin (brand name of Trastuzumab)	II	Patients with IHC 3+HER2 expressing advanced pancreatic cancer or cancer with HER2 gene amplification of stage IVB	17	All patients received trastuzumab at first infusion followed by weekly combined with capecitabine twice daily on days 1–14 of a 3-week cycle.	6.9	Not reported	2.17	NO	[49]
HER1 and HER2 tyrosine kinase inhibitor	Lapatinib	II	Patients with untreated metastatic pancreatic adenocarcinoma	29	Lapatinib and gemcitabine (25); Single agent lapatinib followed by lapatinib and gemcitabine (4)	4±1; The 4 patients who received single agent lapatinib progressed at one month	Not reported	Not reported	NO	[46]
Monoclonal antibody targeting insulin like growth factor-1 receptor (IGF-1R)	Cixutumumab	II	Patients with untreated metastatic pancreatic cancer	116	Cixutumumab+Gemcitabine +Erlotinib (57); Gemcitabine+Erlotinib (59)	7/6.7 (*P*=0.64)	Not reported	3.6/3.6 (*P*=0.97)	NO	[50]
Monoclonal antibody targeting insulin like growth factor-1 receptor (IGF-1R)	Ganitumab	III	Patients with untreated metastatic pancreatic adenocarcinoma	800	Gemcitabine+placebo (322); Gemcitabine+ganitumab 12mg/kg (318); Gemcitabine+ganitumab 20mg/kg (160)	7.2 (6.3–8.2); 7.0 (6.2–8.5) (*P*=0.494) 7.1 (6.4–8.5) (*P*=0.397)	Not reported	3.7 (3.6–4.4); 3.6 (3.4–3.8) (*P*=0.520); 3.7 (3.2–5.0) (*P*=0.403)	NO	[51]
Monoclonal antibody targeting vascular endothelial growth factor A (VEGF-A)	Bevacizumab	II	Patients with previously untreated advanced pancreatic cancer	52	Gemcitabine+bevacizumab	8.8 (7.4–9.7)	29% (17–42%)	5.4 (3.7–6.2)	YES	[52]
Monoclonal antibody targeting vascular endothelial growth factor A (VEGF-A)	Bevacizumab	III	Patients with untreated metastatic pancreatic cancer	535	Gemcitabine+bevacizumab (279); Gemcitabine+placebo (256)	5.8 (4.9–6.6); 5.9 (5.1–6.9); (*P*=0.95)	Not reported	3.8 (3.4–4.0); 2.9 (2.4–3.7); *P*=0.075	NO	[53]
Inhibitor (a recombinant fusion protein) of vascular endothelial growth factor (VEGF)	Aflibercept	III	Patients with metastatic adenocarcinoma of pancreas	546	Gemcitabine+placebo (275); Gemcitabine+aflibercept (271)	7.8 (6.8–8.6); 6.5 (5.6–7.9); *P*=0.2034	25% (18–33%); 21% (13–28%)	3.7 (3.5–4.6); 3.7 (3.5–4.5); *P*=0.8645	NO	[54]
Vascular endothelial growth factor (VEGF) tyrosine kinase inhibitor	Axitinib	III	Patients with metastatic or locally advanced pancreatic adenocarcinoma not amenable to curative resection	632	Gemcitabine+axitinib (316); Gemcitabine+placebo (316)	8.5 (6.9–9.5); 8.3 (6.9–10.3); *P*=0.5436	Not reported	4.4 (4.0–5.6); 4.4 (3.7–5.2); *P*=0.5203	NO	[55]
Inhibitor of tyrosine kinase of VEGFR2, PDGFR-β and B-raf	Sorafenib	II	Patients with untreated advanced pancreatic adenocarcinoma	17	Gemcitabine+sorafenib	4.0 (3.4–5.9)	Not reported	3.2 (1.6–3.6)	NO	[56]
Inhibitor of several receptor tyrosine kinases	Sunitinib	II	Patients with progressive pancreas adenocarcinoma	77	Sunitinib	3.680 (3.055–4.238)	Not reported	1.31 (1.25–1.38)	NO	[57]

### Mutations in the signalling network: directions but chokepoints for targeted therapies at the same time

Targeting a single pathway in PDAC is unlikely to exert impact on disease progression [50]. In other words, various mutations leading to the dysregulation of several signalling pathways have provided possibilities and directions for targeted therapy, but the mutations in diversified pathways circumvent the inhibitory effect of a single target agent, because drug-resistance and the occurrence of tumour resulted from multiple mutations triggering oncogene or inactivating tumour suppressor gene, which could bypass one pathway through another pathway [45,50]. It has been demonstrated that there are multiple crosstalks among the complicated and redundant signal pathways, among which the cross-talks between EGFR and insulin-like growth factor-I (IGF-1R) signalling was studied quite thoroughly [58,59]. EGFR and IGF-1R could interact either through a direct association by mediating the availability of the ligands of each other, or indirectly, via common interaction partners such as G-protein–coupled receptors (GPCR) or downstream signalling molecules [59]. Therefore, blocking or inducing the effect of different dysregulated pathways through some common downstream effectors or combing one targeting drug with others may be a way out of it [45]. Hopefully, this conception has been backed by several preclinical studies that simultaneously blocked two receptors to abrogate downstream signalling shared by both receptors more effectively and thus to gain more inhibitory effect on tumour [60–64]. However, most of these studies were about EGFR and insulin-like growth factor-I (IGF-1R) receptor and some were not focused on pancreatic cancer.

Another possible explanation is that stimulatory signals on tumour progression can be transduced through diverse pathways involving different downstream molecules transducting growth and survival-promoting signals of tumour, such as KRAS, or those in the PI3K/AKT axis signalling pathways, immune to the blocker binding on a certain target [65].

Therefore, the single inhibitor is unable to overcome progression and growth signals from mutations other than the blocked target itself [65]. This explains why most targeted therapies failed to prolong survival.

### Hypovascular TME: divergent circumstances in angiogenesis targeted therapy

Angiogenesis is a crucial part in not only tumorigenesis but also in the process of development in several solid tumours. Vascular endothelial growth factor (VEGF) affects not only the process of angiogenesis of tumour but also the progression and even metastasis of solid tumours. VEGF could be targeted through two kinds of drugs: monoclonal antibody (e.g. bevacizumab [53] and aflibercept [54]) and tyrosine kinase inhibitor (e.g. axitinib [55], sorafenib [56], and sunitinib [66]).

Although prolonged median survival and median PFS were achieved in patients with pancreatic cancer in a phase II clinical trial evaluating the therapeutic effects of the combination of bevacizumab and gemcitabine [52], the same improvement has not been observed in a phase III trial [53], meaning that bevacizumab could not exhibit positive results as expected when combined with gemcitabine at present. Attempts to exploit the therapeutic effect of tyrosine kinase inhibitor on the VEGF pathway suffered similar setbacks. Statistically significant outcomes superior to the control group have not been produced by the combination modalities of gemcitabine and tyrosine kinase inhibitor [55,56].

One convincing reason considered contributing to the ineffectiveness of anti-angiogenic drugs is the hypovascular nature of PDAC, a characteristic widely different from several other solid tumours [67]. On the one hand, the hypovascular nature may enhance drug resistance to anti-angiogenesis treatment by limiting the accessibility of drugs to tumour cells, similar to it does to other drugs. On the other hand, tumours without abundant blood supply as several other solid tumours may not be as susceptible to anti-angiogenesis treatment as those whose occurrence and development are widely dependent on their macroscopically sufficient angiogenesis.

#### Complicated cross-talk between stroma and cancer cells: stimulation and inhibition

The dense stroma is a unique feature of PDAC, strikingly different from several other solid tumours. The role of stroma in PDAC is quite ‘contradictory’, varying from contributing to the progression and drug resistance to impeding tumour cell invasion [19]. Strategies managing to enhance the drug delivery by targeting different components in the abundant fibrotic stroma to alter the fibrotic condition to circumvent the physical barrier have been studied intensively in recent years, among which some produced expected results including increased drug delivery and significant survival improvement, while others did not make any differences or even triggered a progressive evolution of tumour.

Patients with PDAC have high levels of active Hh signalling pathway, as mentioned earlier. Given that, therapeutic approaches targeting the Hh signalling pathway have attracted many interests. However, the results collected so far were not as perfect as expected. Despite that the transient stabilization of disease resulted from transiently increased intra-tumoural vascular density and gemcitabine concentration produced by the co-administration of gemcitabine and Hh signalling pathway inhibitor in a mouse model, two phase II clinical trials failed to improve PFS and overall survival (OS), even one clinical trial of them aiming to testify the promising advantages of modality of gemcitabine and saridegib, inhibitor of the transducer protein SMOOTHENED of Hh signalling pathway, failed due to noticeable and significant tumour progression observed in the combination treatment group [3,68,69].

One possible explanation for the failure of these clinical trials is that the dichotomy of the effect of stroma in PDAC, which means that the stroma could restrain the invasion of cancer cell [18,19,70,71]. Even it has been reported that high stroma density in PDAC is positively correlated with survival [72]. Thus, depletion of stroma regardless of its positive function could lead to a predictable progression of tumour.

Because of the ineffective approaches in depleting the dense stroma through Hh signalling pathway inhibition, various or even contradictory functions of stroma in pancreatic cancer may be considered to bring out the best of stroma-targeting therapies without compromising the advantages of the dense stroma in preventing tumour progression and metastasis. Thus, classifying the various components and recognizing their particular effect on the prevention and promotion of tumour progression and drug resistance to optimally target the stroma without undermining its positive effects should be not only the main focus in targeted therapies but also the key to bring out the best of other drugs administered in the treatment of PDAC clinically in which their delivery has been restrained due to the high density of stroma [3].

## Immune checkpoint inhibitor

Immunotherapy drugs, especially immune checkpoint inhibitors (ICIs), have been the new choice for the treatment of cancer because of their positive effects on several different tumours. The T-cell activation involved with the major histocompatibility complex (MHC) is balanced by the co-stimulation and co-inhibition mechanisms of the immune checkpoint under normal physiological conditions to maintain self-tolerance as well as prevent damage of the normal tissue by the immune system under stress state (Figure 1). However, ligands engaged with the immune checkpoint pathways could be expressed by cancer cells to inhibit the T-cell activation, exhaust the CD8^+^ T cells in the TME, and thus induce T-cell dysfunction and evade immune destruction [73]. Hence, drugs binding to the checkpoints could block the inhibitory effect of tumour cells on the T cell that has accepted the neoantigen presented by the MHC. Unfortunately, ICIs including anti-programmed cell death protein 1 (PD-1), anti-programmed death ligand 1 (PD-L1), or anti-cytotoxic T-lymphocyte-associated antigen 4 (CTLA-4) have not produced promising results in PDAC treatment when they were used as a single agent. Under two prerequisites for the process of presentation of antigen and response of T cell, the presence of distinct neoantigen and robust T-cell infiltration, T-cell activation is then regulated by co-stimulatory and co-inhibitory signalling pathway as mentioned earlier, which could provide proper target for ICI therapy, while PDAC, conventionally deemed to have low immunogenicity, is thought to have neither of the two conditions disappointingly.

### Drug resistance at the gene level: low mutational burden

High tumour mutational burden (TMB) could be a response biomarker for checkpoint inhibitors in tumours such as melanoma and non-small cell lung cancer [74]. In PDAC, ICI therapy did not work when used as a single-agent treatment, except in the case of a rare subset (<2%) of patients with PDAC with microsatellite instability (MSI), which resulted from a mismatch repair (MMR) deficiency [75]. It was because tumours with MMR deficiency or MSI could lead to significantly increased rates of somatic mutations, which could be potentially recognized by antigen-presenting cells (APCs) as neoantigens [76]. Nevertheless, it has been reported that TMB was not a completely reliable biomarker for the response of tumours to immunotherapy [77]. Moreover, it has been revealed in a study using the genomic profiles of 221 PDAC cases extracted from public databases that nearly all PDAC samples harbour potentially targetable neoantigens [78]. Despite the fact that neoantigens in those cases are not as vast as in skin cutaneous melanoma (SKCM), it is enough to create high immunogenicity [75]. Thus, there must be the concept of neoantigen quality, that is, the fitness of a neoantigen, to ensure the antigen presentation by MHC and recognition by T-cell receptor before T-cell response [75].

In some other solid tumours harbouring higher MSI or having more fit neoantigens, T cells can be activated by the neoantigens presented by the APCs and thus express appropriate targets for checkpoint inhibitors. Nevertheless, in PDAC, there is no evident presentation or recognition of neoantigen, let alone factors of co-inhibitory pathway expressed on the T-cell membrane, which is supposed to provide target for checkpoint inhibitors, suggesting that the quality of potential neoantigens of PDAC is much lower than that of SKCM.

### Drug resistance at the TME level: deficiency of tumour-infiltrating lymphocyte infiltration in the suppressive immune microenvironment of PDAC

The most extensively discussed component of the TME contributing to the inefficacy of ICI therapy is tumour-infiltrating lymphocytes (TILs) [79]. According to the histological pattern of TIL, tumours are divided into two categories: T-cell-inflamed (‘hot’ tumours) and non-inflamed (‘cold’) tumours. The former is characterized by the presence of TIL, high density of IFNγ-producing CD8^+^ T cells (cytotoxic T cell), expression of PD-L1 on tumour-infiltrating immune cells, possible genomic instability, and pre-existing anti-tumour immune response. On the contrary, in the latter, T cells are extremely insufficient, and the expression of antigen-presenting machinery markers is extremely deficient [80]. It is suggested that only ‘hot’ tumours could be interfered by a single agent of checkpoint inhibitors by evidence from the preclinical and clinical trials, that is, ICIs could not work without the infiltration of CD8^+^ T cells [80,81]. Therefore, given that most PDAC are defined as ‘cold’ tumours without high TIL levels and required PD-L1 expression, the poor treatment effect of single-agent immune therapies could be explained [82]. The TIL infiltration is limited by (i) the activities of immunosuppressive cells including tumour-associated macrophage (TAM), cancer-associated fibroblasts, myeloid cells, and some deleterious T cells in the TME [83,84]; (ii) fibrotic stroma, which is believed to impede T-cell infiltration, acting as its barrier [85]; and (iii) hypoxia, acting as a primary factor to recruit immunosuppressive cells [75]. CFR-1R^+^ macrophages expressing granulin (GRN) are a dominant type of TAM, which is a principal member of immunosuppressive cells [86,87]. GRN is a secreted glycoprotein of macrophages that stimulates the formation of desmoplastic stroma, correlating with malignancy of various cancer types [88]. The GRN on CSF-1R^+^ macrophages could be strongly induced by CSF-1 secreted by pancreatic cancer cells and then promote the accumulation of myofibroblast during PDAC metastasis [89]. The fibrosis induced by GRN could impede the infiltration of CD8^+^ T cell; therefore, the efficacy of checkpoint inhibitors would be greatly compromised since they only work if CD8^+^ T cells are infiltrated into tumours [89].

### Drug resistance at the molecular level

Recently, a novel mechanism of drug resistance of tumour to ICI therapy has been revealed by Zhao et al. that PD-1 could be co-expressed with PD-L1 on tumour cells and tumour-infiltrating APCs, different from the conventional concept of PD-1 on T cells and PD-L1 on tumour cells and the co-expressed PD-1 could bind to PD-L1 in *cis* on tumour cells [90]. Such interaction then attenuates the ability of PD-L1 to bind T-cell-derived PD-1 in *trans* and, in turn, destroy the canonical PD-L1/PD-1 inhibitory signalling, contrary to the conventional immune escape mechanism unexpectedly. At this time, the exogenous selective inhibitor of PD-1, which should have been combined with PD-1 on T cell, would block tumour cell-derived PD-1 and thus free up tumour cell-derived PD-L1 to bind T-cell-derived PD-1. In other words, tumour cell-derived PD-1 could exhaust the exogenous inhibitor of PD-1 and guarantee the combination of PD-L1 and PD-1 on T cell to inhibit T-cell activation and cytotoxicity [90].

In addition, it has been reported that B7 superfamily member 1 (B7S1) on tumour-infiltrating myeloid cells exerts another co-inhibitory signal on CD8^+^ T cells [91], leading to the dysfunction and exhaustion of effector T cells and contributing to the immune escape of tumour cells. Moreover, this molecule belongs to the third group of B7-CD28 family, while the other two groups contain two noted molecules, CTLA-4 and PD-1 [92]. Thus, to some extent, it explains why the present treatment of single-agent checkpoint inhibitors does not have significant and enduring effects. Additionally, this molecule is expected to be a novel target to block the immune escape of tumour cells and bring out the due effect of checkpoint inhibitors.

## Conclusions

PDAC remains to be a refractory disease and is like to be a tough challenge in the fight against cancer for a long time to come. After years of efforts, the comprehension of the malignant disease has been largely enhanced but not the survival benefit of patients with PDAC. The lack of typical symptoms at early stage, biomarkers with indicative significance for early screening and markers predicting the efficacy of various treatment strategies, and early cancer metastasis have significantly contributed to the dismal prognosis of these patients.

Apart from that, particular mechanisms of drug resistance facing clinicians and investigators in the application and exploration of drugs in the treatment of PDAC, stopping drugs from fully exerting their anti-tumour effects on cancer cells and making the outcome of patients appear to have slim hopes (Table 2). The dense hypovascularized stroma in PDAC widely different from several other solid tumours acts as a dominant factor in limiting the delivery of almost all drugs to tumour cells, which is a key link in severe drug resistance at the TME levels.

**Table 2:** Limitations for PDAC treatment at different levels

Treatment strategy	Dimension of drug resistance	Mechanism of drug resistance
Chemotherapy	TME	Dense stroma
		Hypovascularity
		Hypoxia
	Molecule	Dependence on hENT1
		Up-regulation of cytidine deaminase
		Up-regulation of ribonucleotide reductase
		Up-regulation of thymidylate synthase
	Adverse drug effect	Early termination
Targeted therapy	Diversified mutations	Different mutations sharing same downstream pathway immune to single agent
		Parallel downstream pathway involving different key molecule immune to single agent
	Hypovascularity	Invalidating angiogenesis targeted therapy
	Cross-talk between stroma and cancer cell	Dichotomy of the effect of stroma
Immune checkpoint inhibitors	Gene	Low mutational burden
	TME	Deficiency of tumour-infiltrating lymphocyte infiltration
	Molecule	Co-expressed PD-1 on tumour cells and APCs
		Immune checkpoint apart from CTLA-4 and PD-1 signal

For the superior effect to 5-FU, gemcitabine has remained a corner stone in the chemotherapy of patients with PDAC, but the improvement of clinical outcome is still modest. Apart from the physical barrier, the effect of gemcitabine is compromised by poor membrane permeability, extreme dependence on hENT1, rapid clearance in the bloodstream due to CDA, and change of various enzymes regulating its metabolism. All of them contribute to chemoresistance at the molecular levels.

Chemoresistance at the body levels increases with the applications of novel combinations of chemotherapy aiming to present better anti-tumour effect than single agent gemcitabine. FOLFIRINOX and the doublet of gemcitabine and nab-paclitaxel are the two most prominent agents among them, which have been established as the first-line therapy in clinical practice. However, the accompanied toxicity and adverse effect limit the use of these two regimens to patients with an ECOG performance status of 0–1.

Varieties of dysregulated signalling pathways exert overlapping or distinct effects on tumour proliferation, invasiveness and metastasis and resistance to chemotherapy and radiotherapy, which are not only targets for targeted therapies but also the powerful mechanisms in removing the inhibitory effects of single-agent targeted therapy [45]. Although several preclinical trials have described promising results of targeted therapy, most of them showed disappointing results in phase II or III clinical trials, except erlotinib, an EGFR inhibitor [47]. Stroma-targeting therapy was provided as a new strategy aiming to remove the physical barrier to enhance drug delivery. However, in a clinical trial, co-administering gemcitabine and Hh signalling pathway inhibitor, an evident increased rate of disease progression was observed, completely opposite to what was anticipated, implying that the stroma of PDAC has a kind of particular dichotomy, in which the evil aspect promotes tumour progression, metastasis and chemoresistance, leading to an unfavourable outcome, while the virtuous one inhibits the invasion and delays tumour development [3]. New hopes lie in molecules blocking the common downstream pathway of several synergetic signalling pathways or combination modalities including multiple targeted drugs inhibiting the same pathway or stroma-targeting or cytotoxic drugs. Because of the failures in clinical trials, more biological behaviours of PDAC cells as well as other members in TME are yet to be accurately elucidated.

ICI therapy has already shown promising effects in the treatment of some solid tumours, while single-agent ICI has severely failed in the treatment of PDAC, which is considered to be caused by the low aberrant genomic instability and immunosuppressive microenvironment with rare adaptive immune cells but more immunosuppressive cells, as is traditionally thought to be ‘cold’. Several strategies such as oncolytic viruses, vaccines, and CAR-T-cell therapy have been studied to increase the neoantigen level, enhance the process of presentation and recognition of antigen, and prim T cells for the ICI therapy but are still faced with obstacles.

## Prospect

Altogether, PDAC is a refractory malignant tumour. With decades of investigation, the prognosis of patients with PDAC has been improved modestly. Standard treatment strategies and new emerging approaches with better antineoplastic activity are terribly difficult to play their due role in PDAC treatment due to bottlenecks in different dimensions, varying from clinical drug application to drug resistance mechanism at a molecular level. To circumvent terrible drug resistance, these bottlenecks should be solved, and the poor status quo should be changed or even reversed. Furthermore, personalized combination regimens should be based on deeper and comprehensive understanding of the biological behaviours of cancer cells and various factors in TME might help.

## Abbreviations

APC: antigen-presenting cell
B7S1: B7 superfamily member 1
CTLA-4: cytotoxic T-lymphocyte-associated antigen 4
ICI: immune checkpoint inhibitor
MHC: major histocompatibility complex
MMR: mismatch repair
MSI: microsatellite instability
PD-1: anti-programmed cell death protein 1
PDAC: pancreatic ductal adenocarcinoma
PD-L1: programmed death ligand 1
TIL: tumour-infiltrating lymphocyte
TMB: tumour mutational burden
